# Forecasting incidence of hand, foot and mouth disease using BP neural networks in Jiangsu province, China

**DOI:** 10.1186/s12879-019-4457-6

**Published:** 2019-10-07

**Authors:** Wendong Liu, Changjun Bao, Yuping Zhou, Hong Ji, Ying Wu, Yingying Shi, Wenqi Shen, Jing Bao, Juan Li, Jianli Hu, Xiang Huo

**Affiliations:** 1 Jiangsu Province Center for Diseases Control and Prevention, Nanjing, China; 2 Jiangsu Meteorological Service Center, Nanjing, China

**Keywords:** Hand, foot and mouth disease, Forecasting, BP neural networks

## Abstract

**Background:**

Hand, foot and mouth disease (HFMD) is a rising public health problem and has attracted considerable attention worldwide. The purpose of this study was to develop an optimal model with meteorological factors to predict the epidemic of HFMD.

**Methods:**

Two types of methods, back propagation neural networks (BP) and auto-regressive integrated moving average (ARIMA), were employed to develop forecasting models, based on the monthly HFMD incidences and meteorological factors during 2009–2016 in Jiangsu province, China. Root mean square error (RMSE) and mean absolute percentage error (MAPE) were employed to select model and evaluate the performance of the models.

**Results:**

Four models were constructed. The multivariate BP model was constructed using the HFMD incidences lagged from 1 to 4 months, mean temperature, rainfall and their one order lagged terms as inputs. The other BP model was fitted just using the lagged HFMD incidences as inputs. The univariate ARIMA model was specified as ARIMA (1,0,1)(1,1,0)^12^ (AIC = 1132.12, BIC = 1440.43). And the multivariate ARIMAX with one order lagged temperature as external predictor was fitted based on this ARIMA model (AIC = 1132.37, BIC = 1142.76). The multivariate BP model performed the best in both model fitting stage and prospective forecasting stage, with a MAPE no more than 20%. The performance of the multivariate ARIMAX model was similar to that of the univariate ARIMA model. Both performed much worse than the two BP models, with a high MAPE near to 40%.

**Conclusion:**

The multivariate BP model effectively integrated the autocorrelation of the HFMD incidence series. Meanwhile, it also comprehensively combined the climatic variables and their hysteresis effects. The introduction of the climate terms significantly improved the prediction accuracy of the BP model. This model could be an ideal method to predict the epidemic level of HFMD, which is of great importance for the public health authorities.

## Background

Hand, foot and mouth disease (HFMD) is a common infectious disease caused by various enteroviruses, especially enterovirus 71 (EV-71) and Coxsackie virus A16 (CV-A16) [1]. Recently, enteroviruses other than EV-71 and CV-A16 have been increasing in both mild and severe cases and Coxsackie virus A6 (CV-A6) has been emerging as another predominant serotype in some regions [2]. HFMD mostly affects children under 5 years of age. It is a rising public health problem and has attracted considerable attention worldwide [3–5]. It is especially widespread in Asia-Pacific areas [1] and presents a general increasing incidence in recent decades [6–8]. China is one of the Southeast Asian countries with the most serious HFMD epidemics [9]. It is a Class C notifiable infectious disease in China. Since 2009, the annual incidence of HFMD has never been less than 100 per 100,000, and caused hundreds of deaths in each year. Effective prevention and control of HFMD has become a major challenge in the field of public health [1, 10].

Incidence forecasting of an infectious disease is essential for the public health authorities to better understand the epidemic characteristics and track its seasonal changes in advance. Accurate predicating is a vital basis to optimize decisions and configure resources for preventing and controlling infectious diseases. So, it is of great significance to establish a scientific, appropriate and reliable prediction model and improve the model performance to the best [11, 12]. Recently, some researchers are interested in forecasting the incidence of HFMD, using the liner time series models. For example, an ARIMA (1,0,1, 0,1,0)^12^ model was constructed to forecast the HFMD incidence in Sichuan, China [13]. In another study, multivariable ARIMA models using search engine query data and climate factors as exogenous variables were developed to predict the HFMD epidemic in Guangdong, China [14]. However, the assumption of linearity in many time series events may not be satisfied in practice. The accuracy of the liner forecasting models therefore needs to be improved. Models based on artificial neural networks (ANN) can effectively extract nonlinear relationships in data. They have been widely used in infectious diseases predictions because of their characteristics of robustness, fault tolerance, and adaptive learning ability. As one of the common ANN, back propagation neural networks (BP model) is widely used in many areas, such as economic and engineering. It has also been introduced into forecasting infectious diseases [15, 16]. To date, however, there has been no literature report on using BP model to predict the epidemic of HFMD.

The purpose of this study was to develop an optimal BP model to predict the future trend of HFMD in Jiangsu province, China, with special emphasis on elucidating the effects of meteorological factors as predictors. Meanwhile, the performance of BP model was compared with ARIMA model. It was expected that the findings in this work would be useful for the prevention and control of HFMD.

## Methods

### Data sources

The monthly case numbers of HFMD in Jiangsu province during 2009–2016 were obtained from the National Notifiable Disease surveillance System (http://www.cdpc.chinacdc.cn). The demographic data were collected from the Jiangsu provincial statistics department. And the monthly meteorological data were gained from Jiangsu Meteorological Service Center. The meteorological variables used in this study included rainfall (RF), sunshine duration (SD), relative humidity (RH), atmospheric pressure (AP), minimum temperature (MIN_T), mean temperature (MEAN_T), maximum temperature (MAX_T) and wind velocity (WV).

### BP neural networks

ANN is a family of intelligent methods that mimic the biological neural networks. BP model is one of the most common ANN, developed by Rumelhart and McClelland in 1986 [17]. Since the distinguish performance, BP model has been popularly used in many practical fields including public health [16, 18]. Typical BP model has a three-layer network construction, consisting of an input layer, a hidden layer and an output layer. Each layer consists of a number of neuron nodes. The upper layer and lower layer nodes are connected by the connection weights.

BP model is trained with a back-propagation algorithm, in which the external input information at the input nodes is propagated forward to calculate the outputs. Then the error between the predicted values and the target outputs is propagated backward to modify the connection weights and thresholds. BP model training includes three steps: (1) the forward feeding of the input training pattern, (2) the calculation and back-propagation of the associated error, and (3) the adjustment of the weights and thresholds. Given n nodes in the input layer, m nodes in the hidden layer and one node in the output layer, the outputs of each node in the hidden layer and the output layer are calculated according to the following formulas:

$$ {net}_j=f\left(\sum \limits_{i=0}^n{\omega}_{ij}{x}_i+{b}_j\right)\kern1em \left(\mathrm{i}=0,1,\dots, \mathrm{n};\mathrm{j}=1,2,\dots, \mathrm{m}\right) $$

$$ \hat{y}=f\left(\sum \limits_{j=1}^m{\omega}_j{net}_j+b\right)\kern1em \left(\mathrm{j}=1,2,\dots, \mathrm{m}\right) $$

In the formulas, *net*_*j*_ is the output of the j^th^ node in the hidden layer, *ω*_*ij*_ denotes the connection weight between input node i and hidden node j, *x*_*i*_ the i^th^ input, *b*_*j*_ the threshold of hidden node j, $\hat{y}$ the output of the last layer (i.e., the predicting value), *ω*_*j*_ the connection weight between hidden node j and the output node, b the threshold of output node, f the activation function of a node which is usually a sigmoid function as follow:

$$ f(x)=\frac{1}{1+\exp \left(-x\right)} $$

### ARIMA model

Auto-regressive integrated moving average (ARIMA), also called Box-Jenkins model, is a traditional method to study the time series data [19]. ARIMA model deals with non-stationary time series with a differencing process based on ARMA model. As an extension of ARIMA model, seasonal ARIMA has both non-seasonal and seasonal components [19, 20]. It is denoted as ARIMA(p,d,q)(P,D,Q)^s^ in which p, d, q indicate orders of non-seasonal auto-regression (AR), differencing and moving average (MA) terms; P, D, Q are orders of seasonal AR, differencing and MA, respectively; the superscript s indicates seasonal period (s = 12 in this study). The process of fitting ARIMA model involves four stages. First, the original time series is transformed by logarithmic algorithm, difference or seasonal difference to achieve stationarity. Second, auto-correlation function (ACF) and partial auto-correlation function (PACF) of the stationary time series are calculated and plotted to identify the initial p, q, P, Q parameters. Alternative ARIMA models are established with different model parameters. Third, Akaike information criterion (AIC) and Bayesian information criterion (BIC) are conducted to access the goodness-of-fit of the ARIMA models, the one with the minimum AIC and BIC values is considered as the optimal model. Fourth, Box-Ljung test for the residual series of the optimal model is conducted to determine if the residual series is white noise sequence (*p* > 0.05). Finally, prospective prediction is conducted using the optimal model.

Since the incidence time series of HFMD commonly shows significant cyclical and seasonal patterns [21, 22], ARIMA model was considered to erect the benchmark model. Once the univariate ARIMA model was selected, the multivariate ARIMA model including climate factors as external regressors [23] was further developed. In this study, ARIMA model that incorporates climate factors was referred as ARIMAX.

### Model evaluation

Four models were fitted in this study, BP model with meteorological variables, BP model without meteorological variables, ARIMA model and ARIMAX model. The data between 2009 and 2014 were used as training set to fit models, and data between 2015 and 2016 were used as testing set to evaluate the forecasting accuracy of different models. Root mean square error (RMSE) and mean absolute percentage error (MAPE) were selected as the measures to evaluate the performance of the models, which were calculated as the following formulas:

$$ RMSE=\sqrt{\frac{\sum \limits_{t=1}^n{\left({\hat{y}}_t-{y}_t\right)}^2}{n}} $$

$$ MAPE=\frac{1}{n}\sum \limits_{t=1}^n\frac{\left|{\hat{y}}_t-{y}_t\right|}{y_t} $$

where n means number of real data or predicted values, *y*_*t*_ means real data and ${\hat{y}}_t$ means predicted value.

### Statistical software

All statistical analyses were completed using R software version 3.5.0. Particularly, ARIMA models were performed with R package “forecast” version 8.5. Meanwhile, BP models were constructed with R package “nnet” version 7.3–12.

## Results

### General description

**Fig. 1:**
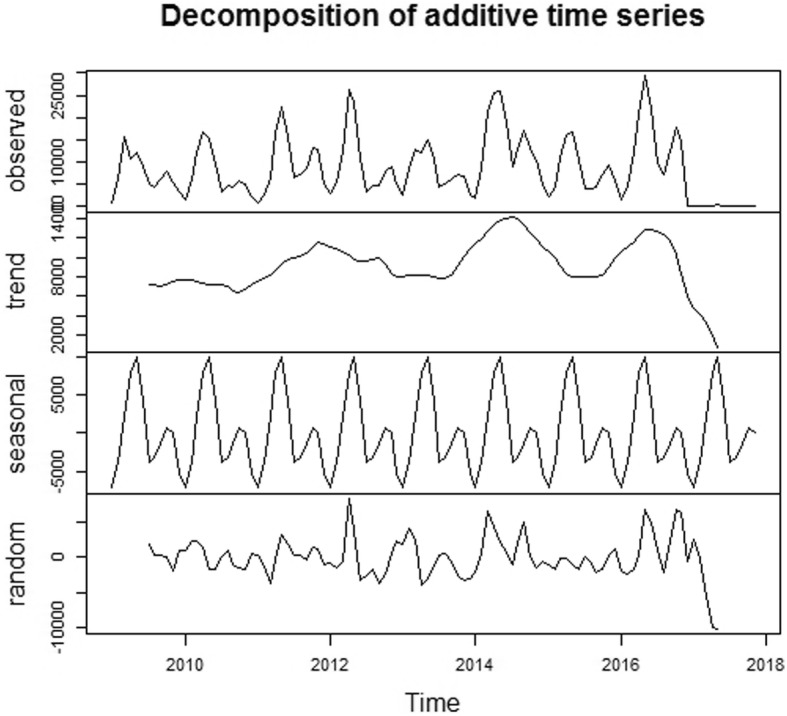
Temporal distribution of HFMD in Jiangsu province, 2009–2016

Totally 917,285 cases were detected during 2009–2016 in Jiangsu province, China, reaching an average annual incidence rate of 145.39 per 100,000. As shown in Fig. 1, the incidence presented no long-term trend in the 8 years. However, there was a distinct seasonality, and two incidence peaks were observed in each year, the higher occurred between April and June, the lower occurred between November and December.

Univariate spearman correlation analysis indicated that all the meteorological factors were significantly associated with the incidence of HFMD, except sunshine duration, relative humidity and wind velocity. Notably, strong correlations were detected among mean temperature, maximum temperature, minimum temperature and atmospheric pressure, with correlation coefficients > 0.9. See Table 1. To avoid multicollinearity, just monthly mean temperature and rainfall were considered in the following models. Further cross-correlation analysis indicated that both mean temperature and rainfall significantly related with the incidence of HFMD at lag 1, with correlation coefficients of 0.235 (*p* = 0.0216) and 0.251 (*p* = 0.0146) respectively. These lagged terms were also considered in the following modelling.

**Table 1:** Spearman correlation coefficients between HFMD and meteorological factors in Jiangsu province, 2009–2016

	HFMD	RF	SD	RH	AP	MIN_T	MEAN_T	MAX_T
RF	0.334^*^							
SD	0.129	−0.142						
RH	0.167	0.752^*^	−0.404^*^					
AP	−0.400^*^	−0.723^*^	− 0.330^*^	−0.471^*^				
MIN_T	0.409^*^	0.741^*^	0.300^*^	0.608^*^	−0.943^*^			
MEAN_T	0.410^*^	0.719^*^	0.334^*^	0.576^*^	−0.947^*^	0.998^*^		
MAX_T	0.409^*^	0.686^*^	0.387^*^	0.526^*^	−0.949^*^	0.990^*^	0.996^*^	
WV	0.045	0.151	0.099	−0.241^*^	−0.175	− 0.033	−0.035	− 0.029

^*^: *p* < 0.05

### Model fitting

#### Multivariate BP model

**Fig. 2:**
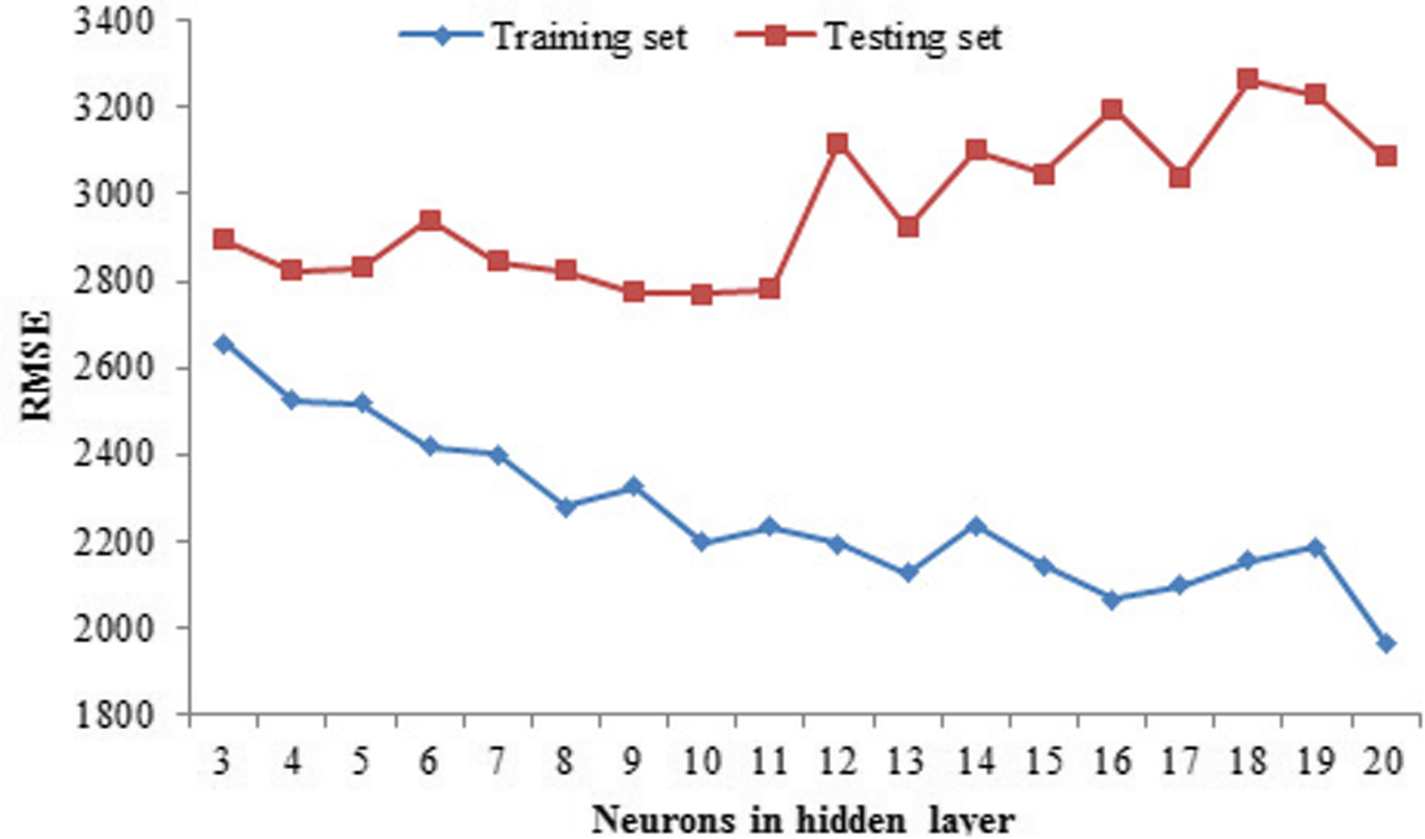
Performance of BP models with different neurons in hidden layer

According to the results of autocorrelation analysis, the HFMD time series presented significant autocorrelation at lag 1–4. Given this, its four lagged terms were considered as predictors in the BP model. Ultimately, eight variables, including the monthly case numbers lagged from 1 to 4 months (× 1-× 4), the monthly mean temperature and rainfall (× 5, × 6), and the one order lagged mean temperature and rainfall (× 7, × 8), were taken as inputs of the BP model. The current monthly case number was taken as output of the model. To determine the number of neurons in the hidden layer, 18 BP models with different neurons in the hidden layer were built, and RMSE was employed to evaluate their performance. As shown in Fig. 2, the model performed better on the training set with more neurons in the hidden layer, which means the more neurons in the hidden layer, the better goodness-of-fit. The prospective prediction accuracy reached the best on the testing set when the number of neurons in the hidden layer was 11. Accordingly, the best BP model structure was determined as 8–11-1, which means there were 8 nodes in the input layer, 11 nodes in the middle layer and one node in the output layer.

#### Univariate BP model

To assess the effects of meteorological factors on the forecasting of HFMD, a BP model without climate variables was constructed, just using the cases numbers lagged from 1 to 4 months (× 1-× 4) as the model inputs. The model fitting process was the same as the former BP model. And the best model structure was determined as 4–4-1.

#### Univariate ARIMA model

The original time series of HFMD achieved stationary after one order seasonal differencing. As shown in Table 2, eight alternative univariate ARIMA models were constructed. The results of Ljung-Box test suggest that all the residual series of these models were white noise sequences. Based on the AIC and BIC, the best-fitting model was determined to be ARIMA(1,0,1)(1,1,0)^12^, with a minimum AIC = 1132.12 and a minimum BIC = 1440.43.

**Table 2:** Selection of the univariate ARIMA model

Model	AIC	BIC	Ljung-Box test	
			Q^a^	p
ARIMA(1,0,1)(1,1,0)^12^	1132.12	1140.43	10.233	0.5272
ARIMA(1,0,0)(1,1,0)^12^	1135.73	1141.96	17.418	0.1432
ARIMA(2,0,1)(1,1,0)^12^	1133.77	1144.16	10.187	0.4422
ARIMA(2,0,0)(1,1,0)^12^	1134.98	1143.29	13.207	0.2944
ARIMA(3,0,1)(1,1,0)^12^	1134.63	1147.09	9.118	0.4455
ARIMA(3,0,0)(1,1,0)^12^	1132.76	1143.15	8.6807	0.5811
ARIMA(4,0,1)(1,1,0)^12^	1136.61	1151.15	8.4172	0.4136
ARIMA(4,0,0)(1,1,0)^12^	1134.64	1147.11	8.6288	0.4917

^a^Q denotes the statistics of Ljung-Box test

#### Multivariate ARIMAX model

Mean temperature (× 5), rainfall (× 6) and their one order lagged terms (× 7 and × 8, respectively) were added into the optimal univariate ARIMA(1,0,1)(1,1,0)^12^ model as exogenous variables, individually or in combination. Accordingly, 15 multivariate ARIMAX models were fitted. As shown in Table 3, these models were all statistically significant (Ljung-Box test *p* > 0.05). ARIMA(1,0,1)(1,1,0)^12^ with one order lagged temperature as external predictor was the optimal ARIMAX model, with a minimum AIC = 1132.37 and a minimum BIC = 1142.76.

**Table 3:** Selection of the multivariate ARIMAX model

Model	AIC	BIC	Ljung-Box test	
			Q^a^	p
ARIMA+×5	1134.05	1144.43	10.679	0.4005
ARIMA+×6	1134.08	1144.47	10.274	0.4347
ARIMA+×7	1132.37	1142.76	10.982	0.3759
ARIMA+×8	1134.07	1144.45	10.272	0.4348
ARIMA+×5 + × 6	1135.97	1148.43	10.903	0.2985
ARIMA+×5 + × 7	1134.01	1146.47	10.406	0.3357
ARIMA+×5 + × 8	1135.97	1148.43	10.85	0.3023
ARIMA+×6 + ×7	1134.21	1146.68	11.149	0.2811
ARIMA+×6 + ×8	1136.06	1148.52	10.28	0.3456
ARIMA+×7 + ×8	1134.00	1146.46	11.144	0.2815
ARIMA+×5 + ×6 + ×7	1135.93	1150.48	10.502	0.2468
ARIMA+×5 + ×7 + ×8	1135.62	1150.16	10.325	0.2587
ARIMA+×5 + ×6 + ×8	1137.94	1152.49	10.926	0.2201
ARIMA+×6 + ×7 + ×8	1135.99	1150.53	11.157	0.2066
ARIMA+×5 + ×6 + ×7 + ×8	1137.61	1154.23	10.294	0.1861

^a^Q denotes the statistics of Ljung-Box test

### Prediction performance comparison

**Fig. 3:**
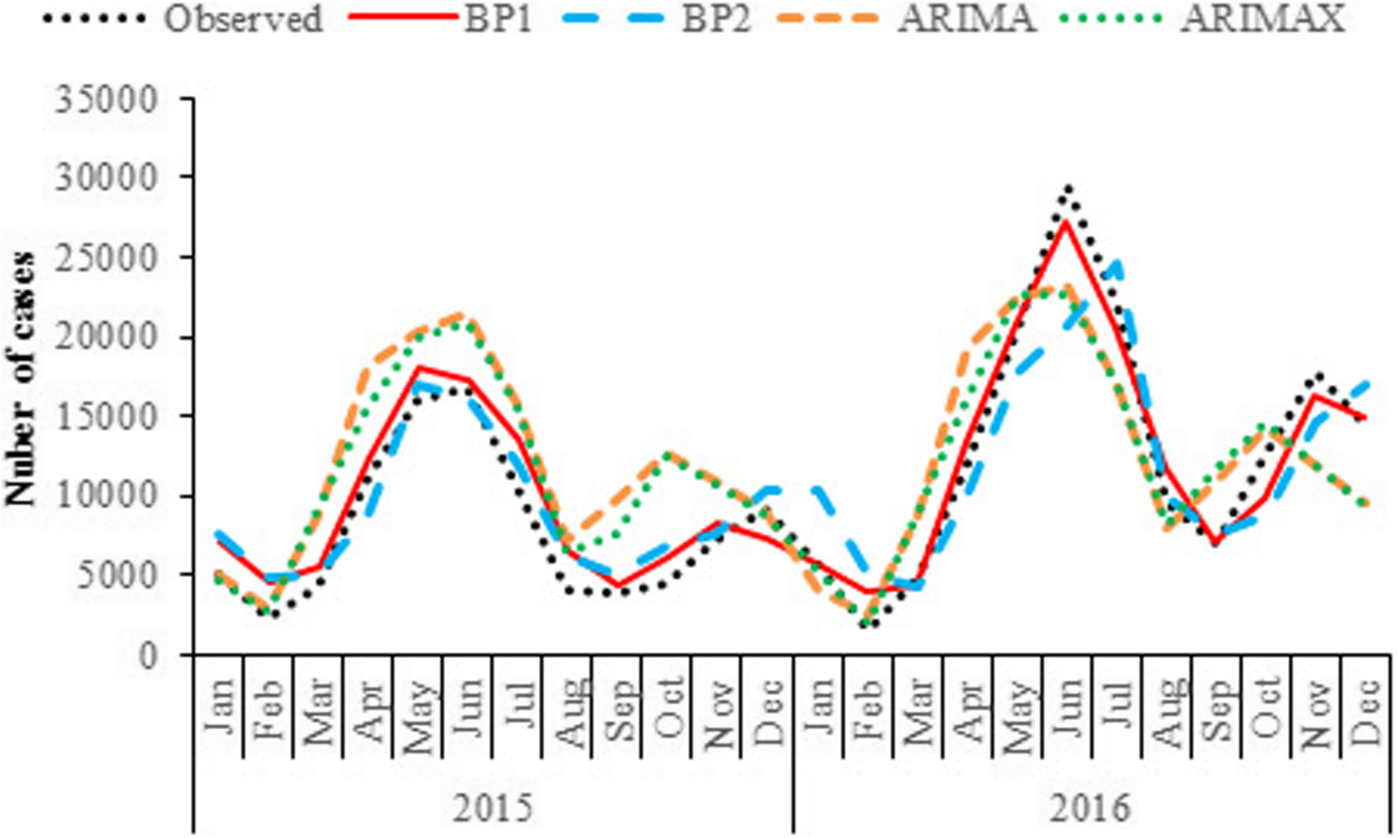
Plot of observed HFMD incidences and predicted values via different models (Note: BP1 means BP model with climate factors, BP2 means BP model without climate factors)

The comparison of the models was summarized in Table 4, and the predicting outputs were displayed in Fig. 3. The multivariate BP model performed the best in both model fitting stage and prospective forecasting stage. The predicted values matched the real HFMD incidences very well, with a MAPE no more than 20%, which suggested that this model would be able to accurately estimate the prevalence and seasonal fluctuation of HFMD. The BP model without climate variables had much higher RMSE and MAPE than the multivariate BP model on both training set and testing set. Its forecasting performed well in 2015 but presented a high error in 2016. The performance of the multivariate ARIMAX model was similar to that of the univariate ARIMA model. Both performed much worse than the two BP models. They could not accurately predict the real data in the study area, with a high MAPE near to 40%.

**Table 4:** Comparison of the four models

model	RMSE		MAPE	
	Training set	Testing set	Training set	Testing set
Multivariate BP	2125.68	2653.73	16.59	18.57
Univariate BP	3055.32	2981.11	20.46	28.55
ARIMA(1,0,1)(1,1,0)^12^ + ×7^a^	3313.60	4476.06	26.11	36.43
ARIMA(1,0,1)(1,1,0)^12^	3377.48	4476.39	28.02	36.67

^a^×7 means one order lagged mean temperature

## Discussion

Accurately identifying the epidemic trend in advance is of critical importance for infectious diseases prevention and control. As HFMD is a common infectious disease throughout the world, modeling its epidemic has been concerned and actively studied in recent years. Some researchers have put forward different prediction methods for HFMD. For examples, Yu et al. [24], developed a new hybrid model with ARIMA and nonlinear auto-regressive neural network. Zhong et at [25]., employed XGBoost, one of the machine learning methods, to forecast HFMD with multiple environmental factors.

In this study, an optimized multivariate BP model with meteorological factors was constructed. This model presented a satisfactory accuracy in forecasting the HFMD incidences in Jiangsu province, China. It reached a MAPE less than 20% in the prospective forecasting stage and accurately estimated the seasonal fluctuation of HFMD in the next 24 months. The predictive performance is much better than that in many similar studies. It may serve as a reliable tool for the public health authorities in the practice of HFMD prevention and control. Notably, BP model has a risk of over-fitting, which is a critical issue that usually leads to poor generalization [26]. In this work, it was observed that the accuracy of prospective predication getting worse when the neurons in the hidden layer was more than 11, which suggested that too many neurons in hidden layer maybe causes severe over-fitting. Hence, how to determine an optimized model structure is an important issue. Unfortunately, it is still controversial. In this study, this work was completed based on MAPE, the BP model with the minimum MAPE on testing set was selected as the best optimal model.

A substantial studies have proposed that infectious diseases are climate sensitive [27–29]. Climatic factors may influence the survival and spread of infectious pathogens in the environment, the host susceptibility and exposure probability [30–32]. The effects of meteorological factors, such as temperature, rainfall and relative humidity, on the epidemics of HFMD have attracted considerable concerning recently [22, 33]. Song et al. [34], developed a seasonal ARIMA model with lagged precipitation as predictor to forecast the incidence of HFMD. Unfortunately, the model did not present a satisfactory performance. Similarly, the ARIMAX model we developed using lagged temperature as predictor did not achieve a good enough accuracy for practical application. And the introduced climate variable did not improve the performance of the ARIMA model. It may be due to two reasons. Firstly, ARIMA model is essentially a linear method. However, meteorological factors were proved to be non-linearly associated with the epidemic of HFMD, so ARIMA model is inappropriate to fit the relationships between predictors and HFMD incidence. Besides, data size is not sufficient for the model to fully extract the underline pattern contained in data. Consequently, the model could not achieve a satisfied predictions. Zhao et al. [35], also constructed an ARIMA model with temperature as predictor based on the data in Huainan City, China. It presented a well goodness-of-fit. However, its extrapolated predictive capability was not explained. Consequently, its practical application value is doubtful. In this study, BP neuron network was employed to forecast the HFMD incidence with monthly average temperature, rainfall and their lagged terms as predictors. This model performed much better than the BP model without climatic variables, which suggested that climate factors can improve the prediction effect. Meanwhile, we also found that both of the two BP models performed much better than the ARIMA models. This may indicate that BP model is more suitable than ARIMA model to predict the HFMD incidence in the study region.

It is worth mentioning that the multivariate BP model developed in this study achieved accurate estimations of the HFMD incidences in the next 24 months. Thus, it could be used to predict the medium to long term epidemic level of HFMD, which is of great important for the public health authorities. As shown in Fig. 3, given the whole test set, the BP model without climate variables performed relatively poor. Interestingly, the first few predicted values matched the real incidences very well. It suggested that this model may have the potential to be used for short-term forecasting, which is necessary to be further verified in practice.

Some limitations need to be mentioned. First, the epidemic of HFMD is affected by many factors, including natural and social environmental factors, etiological factors, and so on. In this study, just meteorological variables were considered to improve the predication ability. Other factors associated with HFMD may also be used as good predictors, which deserves progressive studies. Second, because some mild cases might use home therapies, and some cases with atypical symptoms may be misdiagnosed, so the data reported may underestimate the HFMD incidence, which may affect the precision of the predictions. Third, the optimal BP model was constructed based on the data in Jiangsu province, China, generalizability of our findings to other regions with different epidemic characteristics of HFMD and climate situations might not be straightforward. But the use of the BP model incorporating climate factors in the detection and prediction of HFMD may provide an opportunity for re-allocating healthcare resources more efficiently in other regions or countries. Besides, similar to many other neural network models, BP model can not explain the specific association between risk factors and disease.

## Conclusion

In this study, four models were constructed to forecast the incidence of HFMD in Jiangsu province, China. The BP models performed much better than the ARIMA models. The introduction of mean temperature, rainfall and their one order lagged terms significantly improved the prediction accuracy of the BP model. On the contrary, neither the univariate ARIMA model nor the multivariate ARIMAX model achieved satisfactory prediction accuracy. The climate factors did not optimize the performance of the ARIMA model. In general, the multivariate BP model comprehensively combined the autocorrelation of the independent, the climatic variables and their hysteresis effects. It is an ideal method to predict the HFMD epidemic, which has a good prospect of practical application.

## Data Availability

The datasets used in this study are available from the corresponding author on reasonable request.

## Abbreviations

ACF: Auto-correlation function
AIC: Akaike information criterion
ANN: Artificial neural networks
AP: Atmospheric pressure
ARIMA: Auto-regressive integrated moving average
ARIMAX: ARIMA model that incorporates climate factors
BIC: Bayesian information criterion
BP: Back propagation neural networks
CV-A16: Coxsackie virus A16
CV-A6: Coxsackie virus A6
EV-71: Enterovirus 71
HFMD: Hand, foot and mouth disease
MAPE: Mean absolute percentage error
MAX_T: Maximum temperature
MEAN_T: Mean temperature
MIN_T: Minimum temperature
PACF: Partial auto-correlation function
RF: Rainfall
RH: Relative humidity
RMSE: Root mean square error
SD: Sunshine duration
WV: Wind velocity
